# Supplementary material for: Resilience in the Storm: Impacts of Changed Daily Lifestyles on Mental Health in Persons with Chronic Illnesses under the COVID-19 Pandemic
Source: Int J Environ Res Public Health. 2021 May 30;18(11):5875. doi: 10.3390/ijerph18115875 (PMC8198084; doi:10.3390/ijerph18115875)
Supplement: Supplementary file 1 [file ijerph-18-05875-s001.zip › ijerph-1195830-supplementary.pdf]

Table S1. Results of ANOVAs with post-hoc analysis.

	Change of Daily Lifestyles	Mean	SD	P-values of omnibus effects	P-values of Post-hoc tests with Tukey's adjustment		
					2	3	4
	**Age**						
	WHO5			0.083			
1	<55	43.5	20.8		0.163	0.086	
2	55-64	48.7	23.4			0.953	
3	65 or above	49.5	22.4				
	**DASS-21 Depression**			0.039			
1	<55	7.8	9.2		0.155	0.032	
2	55-64	8.7	8.4			0.765	
3	65 or above	6.0	8.0				
	**DASS-21 Anxiety**			0.019			
1	<55	8.8	9.3		0.483	0.016	
2	55-64	8.9	7.2			0.193	
3	65 or above	6.8	7.1				
	**DASS-21 Stress**			0.008			
1	<55	12.0	9.9		0.115	0.005	
2	55-64	12.6	8.9			0.450	
3	65 or above	8.7	8.9				
	**CD-RISC**			<0.001			
1	<55	27.7	8.3		0.029	0.000	
2	55-64	25.6	7.8			0.296	
3	65 or above	28.4	7.5				
	**Sex**						
	**WHO5**			0.168			
1	Female	46.4					
2	Male	49.6					
	**DASS-21 Depression**			0.232	0.770		
1	Female	7.7					
2	Male	7.5					
	**DASS-21 Anxiety**			0.005			
1	Female	8.8					
2	Male	6.8					
	**DASS-21 Stress**			0.927			
1	Female	11.0					
2	Male	10.9					
	**CD-RISC**			0.642			
1	Female	26.8	7.7				
2	Male	27.2	8.1				
	**Disabling effect of chronic conditions**						
	**WHO5**			0.101			
1	1 type	48.7			0.995	0.415	0.702
2	2 types	48.0				0.593	0.779
3	3 types	43.0					0.999
4	4 types	42.0					
	**DASS-21 Depression**			0.421	0.004		
1	1 type	6.6			0.312	0.001	1.000

2	2 types	8.3				0.074	0.871
3	3 types	11.9					0.147
4	4 types	6.5					
	**DASS-21 Anxiety**			0.000			
1	1 type	7.2			0.532	0.008	0.174
2	2 types	8.3				0.170	0.483
3	3 types	11.0					0.999
4	4 types	11.3					
	**DASS-21 Stress**			0.003			
1	1 type	9.8			0.134	0.081	0.514
2	2 types	12.1				0.863	0.970
3	3 types	13.4					1.000
4	4 types	13.3					
	**CD-RISC**			0.390			
1	1 type	27.3	7.9		0.891	0.446	0.957
2	2 types	26.7	7.9			0.806	0.861
3	3 types	25.4	8.0				0.594
4	4 types	28.4	5.2				
	**Exercise**						
	WHO5			0.037			
1	No	43.6	22.1		0.997	0.484	0.596
2	Reduce	44.6	21.3			0.048	0.277
3	Same	51.0	22.9				1.000
4	Increase	50.8	24.3				
	**DASS-21 Depression**			0.038			
1	No	7.8	9.2		0.970	0.793	0.999
2	Reduce	8.7	8.4			0.022	0.973
3	Same	6.0	8.0				0.385
4	Increase	8.1	9.4				
	**DASS-21 Anxiety**			0.056			
1	No	8.8	9.3		1.000	0.616	0.950
2	Reduce	8.9	7.2			0.036	0.751
3	Same	6.8	7.1				0.792
4	Increase	7.8	7.2				
	**DASS-21 Stress**			0.002			
1	No	12.0	9.9		0.992	0.403	0.914
2	Reduce	12.6	8.9			0.001	0.423
3	Same	8.7	8.9				0.601
4	Increase	10.5	8.9				
	**CD-RISC**			0.009			
1	No	27.7	8.3		0.649	0.981	0.999
2	Reduce	25.6	7.8			0.008	0.184
3	Same	28.4	7.5				0.990
4	Increase	28.0	8.2				
	**Outdoor**						
	**WHO5**			0.000			
1	No	59.5	29.2		0.224	0.865	0.999
2	Reduce	44.4	21.6			0.003	0.021
3	Same	53.2	22.6				0.580
4	Increase	60.8	19.9				
	**DASS-21 Depression**			0.232			
1	No	6.6	6.9		0.946	1.000	1.000

2	Reduce	8.2	8.3			0.191	0.909
3	Same	6.3	8.6				0.997
4	Increase	6.8	10.5				
	**DASS-21 Anxiety**			0.212			
1	No	10.9	10.8		0.764	0.431	0.807
2	Reduce	8.4	7.3			0.276	0.999
3	Same	6.9	7.4				0.925
4	Increase	8.1	5.7				
	**DASS-21 Stress**			0.020			
1	No	9.6	10.5		0.897	0.996	0.987
2	Reduce	11.9	9.0			0.021	0.413
3	Same	8.9	9.0				0.995
4	Increase	8.3	7.7				
	**CD-RISC**			0.000			
1	No	30.8	9.0		0.649	0.981	0.999
2	Reduce	25.8	7.6			0.008	0.184
3	Same	29.1	7.9				0.990
4	Increase	31.1	6.9				
**Utilization of NGO services**							
	**WHO5**			0.065			
1	No	48.6	23.4		0.739	0.550	0.900
2	Reduce	45.7	22.0			0.072	0.532
3	Same	53.9	22.9				0.993
4	Increase	52.4	21.2				
	**DASS-21 Depression**			0.421			
1	No	8.5	9.8		0.936	0.474	0.691
2	Reduce	7.8	8.2			0.628	0.830
3	Same	6.3	7.8				1.000
4	Increase	6.2	8.5				
	**DASS-21 Anxiety**			0.007			
1	No	8.8	7.8		0.986	0.225	0.024
2	Reduce	8.5	7.3			0.205	0.021
3	Same	6.3	7.3				0.520
4	Increase	3.8	4.3				
	**DASS-21 Stress**			0.386			
1	No	10.6	9.9		0.899	0.914	0.891
2	Reduce	11.5	8.8			0.517	0.638
3	Same	9.6	9.0				0.996
4	Increase	9.1	9.5				
	**CD-RISC**			0.013			
1	No	27.0	8.0		0.908	0.891	0.058
2	Reduce	26.3	7.8			0.486	0.010
3	Same	28.0	8.1				0.229
4	Increase	31.8	6.5				
**Utilization of SHO services**							
	**WHO5**			0.003			
1	No	46.5	22.3		0.977	0.032	0.122
2	Reduce	45.5	22.1			0.011	0.075
3	Same	57.1	22.4				0.991
4	Increase	59.1	19.8				
	**DASS-21 Depression**			0.414			
1	No	7.9	9.1		1.000	0.613	0.704

2	Reduce	8.0	8.2			0.559	0.675
3	Same	6.1	8.0				0.996
4	Increase	5.6	7.1				
	**DASS-21 Anxiety**			0.214			
1	No	8.3	7.5		1.000	0.203	1.000
2	Reduce	8.3	7.2			0.179	1.000
3	Same	5.8	6.8				0.579
4	Increase	8.4	8.3				
	**DASS-21 Stress**			0.085			
1	No	11.4	9.7		1.000	0.090	0.885
2	Reduce	11.4	8.7			0.074	0.876
3	Same	7.7	8.1				0.867
4	Increase	9.7	9.2				
	**CD-RISC**			0.035			
1	No	26.8	8.5		0.956	0.132	0.476
2	Reduce	26.3	7.5			0.052	0.337
3	Same	29.7	6.9				1.000
4	Increase	29.6	6.4				
	**Contact with others**						
	**WHO5**			0.000			
1	No	37.1	23.0		0.758	0.065	0.666
2	Reduce	43.8	22.4			0.000	0.965
3	Same	54.1	21.2				0.124
4	Increase	45.6	22.3				
	**DASS-21 Depression**			0.002			
1	No	12.7	10.4		0.447	0.049	0.108
2	Reduce	8.9	8.6			0.007	0.275
3	Same	6.0	8.2				0.996
4	Increase	6.3	6.7				
	**DASS-21 Anxiety**			0.037			
1	No	10.5	9.3		0.886	0.354	0.750
2	Reduce	8.9	7.5			0.037	0.911
3	Same	6.8	6.9				0.753
4	Increase	8.0	7.3				
	**DASS-21 Stress**			0.000			
1	No	15.6	8.9		0.729	0.051	0.234
2	Reduce	12.8	9.3			0.000	0.239
3	Same	8.5	8.6				0.797
4	Increase	9.9	7.2				
	**CD-RISC**			0.000			
1	No	21.4	12.0		0.286	0.005	0.152
2	Reduce	25.6	7.4			0.000	0.768
3	Same	29.3	7.7				0.258
4	Increase	26.8	7.1				
	**Gathering with others**						
	**WHO5**			0.026			
1	No	33.8	19.3		0.286	0.039	0.291
2	Reduce	47.1	22.4			0.119	0.751
3	Same	56.1	20.1				0.992
4	Increase	60.0	40.0				
	**DASS-21 Depression**			0.138			
1	No	13.2	10.3		0.205	0.168	0.215

2	Reduce	7.6	8.4			0.925	0.700
3	Same	6.7	8.9				0.828
4	Increase	2.3	4.0				
	**DASS-21 Anxiety**			0.559			
1	No	11.3	8.9		0.551	0.481	0.921
2	Reduce	8.0	7.3			0.956	1.000
3	Same	7.3	7.3				0.998
4	Increase	8.2	11.3				
	**DASS-21 Stress**			0.101			
1	No	15.9	9.8		0.369	0.238	0.110
2	Reduce	11.0	9.0			0.813	0.350
3	Same	9.5	9.1				0.550
4	Increase	2.3	4.0				
	**CD-RISC**			0.013			
1	No	19.8	10.9		0.034	0.007	0.318
2	Reduce	26.9	7.6			0.329	0.981
3	Same	29.3	8.8				0.999
4	Increase	28.7	7.6				
